# A study of snake bite among children presenting to a paediatric ward in the main Teaching Hospital of North Central Province of Sri Lanka

**DOI:** 10.1186/1756-0500-7-482

**Published:** 2014-07-29

**Authors:** Roshini Kilanthi Karunanayake, Dissanayake Mohottalage Randima Dissanayake, Aranjan Lionel Karunanayake

**Affiliations:** 1 Teaching Hospital, Sri Jayawardenapura, Sri Lanka; 2 Teaching Hospital, Chilaw, Sri Lanka; 3 College of Medicine, Nursing & Health Sciences, National University of Fiji, Suva, Fiji

**Keywords:** Snake bite, Pediatrics, Venomous, Sri Lanka

## Abstract

**Background:**

Snake bite is a common problem in the North Central province of Sri Lanka. Common krait (*Bungarus careuleus*), Ceylon krait (*Bungarus ceylonicus*), Cobra (*Naja naja*), Russell’s viper (*Daboia russelii*), Saw-scaled viper (*Echis carinatus*) and Hump-nosed pit viper (*Hypnale hypnale*) are the six species of venomous land snakes in Sri Lanka. A significant number of adults and children are bitten by snakes every year. However, the majority of research studies done in Sri Lanka and other countries show adults bitten by snakes and studies describing children bitten by snakes are very sparse.

**Methods:**

A descriptive cross sectional study was performed in the Teaching Hospital Anuradhapura in the North Central Province of Sri Lanka from May 2010 to 2011 May to describe the characteristics associated with cases of snake bite.

**Results:**

There were 24 males and 20 females. The highest numbers of bites (48%) were in the range of ages 6-12 years. The majority of the bites occurred between 6 pm to 6 am (59%).The foot was the most common bitten site (48%). Out of all the venomous bites, the Hump-nosed pit viper (*Hypnale hypnale*) accounted for the highest number (44%) and Russell’s viper (*Daboia ruselii*) accounted for the second highest number (27%). A significant number of venomous bites occurred indoors while sleeping (22%). Antivenom serum was given to (39%) of venomous bites. Deaths occurred in (11%) of the venomous bites.

**Conclusions:**

Hump-nosed pit viper (*Hypnale hypnale*) accounted for the highest number of venomous bites. Majority of the bites occurred between 6 pm and 6 am. Foot was the most common bitten site. A significant number of venomous bites occurred indoor while sleeping. Antivenom serum was given to a significant number of venomous bites. Educating the public on making their houses snake proof and using a torch when going out during night time will help in the prevention of getting bitten by snakes.

## Background

Every year a large number of snake bite envenomations occur worldwide [1]. According to worldwide statistics, 5% of snake bite victims end in fatalities [2]. The highest number of snake bites is recorded from Asia, Southeast Asia and Sub-Saharan Africa [3]. South Asia is the world’s most heavily affected region [4]. The burden of snake bite is highest in developing countries [5]. Bites are more frequent in young men [6].

According to a study done in Bangladesh, the majority of the bites have occurred while people were at work (71%) and the most common bite site were the lower limbs [7]. In North India most of the bites (61%) occurred when patients were asleep [8]. A study done in Kangar district Malaysia shows that prevalence of snake bites among 0-9 yrs (7%), 10-19 yrs (33%), 20-29 yrs (17%), 30-39 yrs (14%), 40-49 yrs (6%), 50-59 yrs (11%), 60-69 yrs (8%) and more than 70 yrs were 5% [9].

Annually around 33,000 venomous snake bite victims are reported from government hospitals in Sri Lanka (4). In the year 2000, 0.5% of snake bite victims admitted to regional hospitals in Sri Lanka ended in death [10]. In Sri Lanka, the Common krait (Bungarus careuleus) Ceylon krait (*Bungarus ceylonicus)*, Cobra (*Naja naja*), Russell’s viper (*Daboia russelii*) and Saw-scaled viper (*Echis carinatus*) are considered potentially deadly [11].

Blisters and local necrosis are common especially in Cobra (*Naja naja*) bites [6].

Russell’s viper (*Daboia russelii*) bites cause many problems such as bleeding and neurological complications [12]. Acute renal failure is a major complication of viper bites [13]. It is believed that Russell’s viper (*Daboia russelii*) bites rarely cause myotoxicity [14].

Hump-nosed pit viper (*Hypnale hypnale*) comes under a moderately venomous group. Their bites cause pain and induration at the bite site and local haemorrhagic blister formation [15]. Hump-nosed pit vipers (*Hypnale hypnale*) can cause renal tubular acidosis [16]. A rare death has been reported in Sri Lanka due to acute kidney injury after a Hump-nosed pit viper (*Hypnale hypnale*) bite [17].

Saw-scaled viper (*Echis carinatus*) bites can give rise to local swelling and coagulopathy [18]. They inhabit the coastal areas of the dry zone [19]. A study done by Kularatne et al. [20] in Jaffna peninsula Sri Lanka shows that 15% of bite victims were school children.

Common krait (*Bungaruscareuleus*) bites can give rise to burning body pains, speech and swallowing difficulties and respiratory failure [21].

Majority of studies done in the world have been done on adult snake bite victims. Very few studies survey paediatric snake bite victims and they are surveyed along with adult snake bite victims. Paediatric victims in those studies account only for a small percentage of the victims. Studies done solely on paediatric victims are very sparse. Bite circumstances, the time taken to admit patient to the hospital, and the outcome of paediatric snake bite victims can differ significantly from adult snake bite victims. Therefore, this study was carried out in the Teaching Hospital Anuradhapura district on paediatric snake bite victims.

## Methods

A descriptive cross sectional study was done at the main Teaching Hospital in Anuradhapura district to describe the characteristics of snake bite according to the locale site, time, activity at the time of bite, site of bite, species of snake that caused the bite, clinical features of snake bite victims, first aid provided at home, time taken to admit to the hospital, and the medical management at the hospital.

Anuradhapura district is located 200 km away from the capital of Sri Lanka. All children admitted with a history of snake bite to the main paediatric ward over a period of one year from 2010 May to 2011 May were invited to participate in the study. For data collection, a structured, interviewer administered questionnaire was used. The questionnaire included questions to cover the basic demographic data such as age, sex, geographic location, level of education etc. It also had questions with regards to (time, place, site of bite, type of activity done at the time of bite and species of snake that caused the bite, clinical features, and first aid given at home prior to admission to the teaching hospital). For identification of snakes, patients were shown pictures of snakes to see whether they could select the snake that caused the bite. On some occasions snakes were brought to the ward either killed or alive inside a glass bottle. Then using the help of pictures, the snakes were identified. The pictures of different snake species have been provided to the government hospitals by the Sri Lanka Medical Association.

The snakes that are capable of causing life threatening complications were classified as venomous snakes and the snakes that could cause signs of mild envenoming but without life threatening complications were classified as mild venomous and the snakes that do not cause envenoming were classified as nonvenomous [4]. The bites that caused signs of nephrotoxicity, neurotoxicity, coagulopathy, breathing difficulties, and extensive swelling of the limb (affecting more than half of the bitten limb) were categorized as severe bites. The bites that caused features such as pain, fang marks and swelling of the affected limb (less than half of the affected limb) were named moderately severe bites and the bites that caused pain and fang marks but no signs of envenomation were classified as mildly severe bites [4]. A separate data extraction form was used to extract the following data from the Bed Head Ticket (BHT); date and time of admittance to the ward, discharge date from the hospital, management given at the hospital and the outcome. The management of the patient was performed in the manner such as a patient would be routinely managed in the ward. When antivenom was used, it was administered under supervision with care and when adverse reactions occurred; the patient was resuscitated as per the guidelines followed in the ward. All participants in the study were given advice on first aid that should be given to a snake bite victim and how to prevent being bitten by snakes. The data was entered to a data base and analyzed using the SPSS statistical package.

### Ethical considerations

All children admitted to the paediatric ward were 12 years of age or less. Hence, the written consent to participate in the survey was obtained from the child’s parent or/guardian. The detailed interview was done prior to discharging the patient and not in the acute stage. When a death took place, the guardian’s emotional state was taken into consideration before the interview. Ethical approval to conduct the study was obtained from the ethical review committee of the College of Paediatricians of Sri Lanka.

## Results

There were 27 males and 23 females. Out of these, six were mistaken to have been bitten by snakes but were in fact bitten by centipedes (50%), black ants (33%) and scorpions (17%). The highest number of snake bite victims 21 (48%) were in the age group 6-12 years and lowest 9 (21%) were in the <1 year age group. Envenomed bites were caused by venomous and mildly venomous snakes and a few of the unidentified snakes. Only the venomous species caused the bites that resulted in severe envenomation. Hump-nosed pit viper (*Hypnale hypnale*) accounted for the highest number of venomous bites 8 (44%) (Table 1). Antivenom serum (AVS) had to be administered in 39% of venomous bites (Table 2). Deaths accounted for 2 (5%) of the cases (Table 2). Both were due to Common krait (*Bungarus careuleus*) bites. In our study the three krait bites were caused by *Bungarus careuleus* species. Swelling (59%) and bleeding (48%) were the most common symptoms for these patients (Table 3). Lower limbs were the most common site of bite (73%) and in (7%) of patients the site of bite was in the region of face and scalp (Table 3). The majority of cases (64%) took 1-4 hours to reach the teaching hospital (Table 4). Reassurance (80%) and immobilization (75%) were the most common first aid types given to these patients (Table 4). In our study snake bite victims were children, and they were carried by their parents until they got into the vehicle and were transported to the hospital ward. No pressure bandages or any other total body immobilizations were done. A tourniquet was used in (9%) of cases (Table 4). The majority of the bites 26 (59%) occurred between 6 pm to 6 am (Table 5). A significant number of venomous bites occurred indoors 4 (22%) while sleeping (Table 6).

**Table 1:** Snake species, number and percentage of bites

**Type of snake**	**Number**	**%**
*Venomous*		
Russell’sviper (*Daboiarusselii*)	5	12
Krait (*Bungaruscareuleus*)	3	7
Cobra (*Najanaja*)	1	2
Saw Scaled viper (*Echiscarinatus*)	1	2
Hump-nosed pit viper (*Hypnalehypnale*)	8	18
*Mild venomous*		
Mapila (*Boigaceylonensis*)	4	9
Wolf snake (*Lycodonaulicus*)	1	2
*Non venomous*		
Asian rat snake (*Piyas mucosa*)	1	2
Watersnake (*Xenochrophisasperrimus)*	2	5
Unidentified	18	41
Total	44	100

**Table 2:** Venomous snake species, type of treatment, length of stay and the outcome of snake bite patients at the Teaching hospital Anuradhapura

**Snake species**	**Total number**	**Type of treatment given**	**Number**	**%**	**Outcome**	**Number**	**%**
Hump-nosed pit viper (*Hypnalehypnale)*	8	Symptomatic treatment	8	100	Discharged in ≤ 3 days	8	100
Russel’s viper (*Daboiarusselii)*	5	Antivenom Symptomatic treatment	4	80	Discharged in ≤ 7 days	4	80
			1	20	Discharged in ≤ 3 days	1	20
Saw-scaled viper *(Echiscarinatus*)	1	Antivenom & wound treatment	1	100	Discharged in ≤ 7 days	1	100
Cobra (*Najanaja)*	1	Antivenom & wound treatment	1	100	Discharged in ≤ 7 days	1	100
Common krait (*Bungaruscareuleus)*	3	Antivenom Symptomatic treatment	1	33.3	Death	2	67
			1	33.3	Discharged in ≤ 3 days	1	33
		Death in ITU	1	33.3			

**Table 3:** Site of bite and symptoms and signs

**Site of bite**	**%**	**Symptoms/signs**	**%**
Feet	48	Swelling	59
Legs	25	Bleeding	48
Arms	11	Ptosis	5
Hands	9	Blisters and Necrosis	5
Face	5	Cardiac arrest	2
Scalp	2	Disseminated Intravascular coagulation (DIC)	2
		Abdominal pain	9
		Vomiting	7
		Drowsiness	14

**Table 4:** Types of first aid given and time taken to reach the teaching hospital Anuradhapura

**Types of first aid given**	**%**	**Time taken to reach hospital**	**%**
Reassurance given	80	< 1 hr	18
Immobilization done	75	1-4 hrs	64
Washed with soap and water	62	>4 hrs	18
Tourniquet applied	9		
Sucked the wound	0		
Herbal Application	5		
Native treatment	2		

**Table 5:** Snake species and the time of bite

**Snake species**	**Total number**	**Time of bite between (6 am - 6 pm)**	**%**	**Time of bite between (6 pm - 6 am)**	**%**
		**Number**		**Number**	
Hump-nosed pit viper (*Hypnalehypnale)*	8	6	75	2	25
Russel’s viper (*Daboiarusselii)*	5	1	20	4	80
Saw-scaled viper *(Echiscarinatus*)	1	-	-	1	100
Cobra (*Najanaja)*	1	-	-	1	100
Common krait (*Bungaruscareuleus)*	3	1	33	2	67

**Table 6:** Snake species, place of bite and the activity done at the time of bite

**Snake species**	**Total number**	**Place of bite (Outdoor)**	**%**	**Place of bite (Indoor)**	**%**	**Activity done at the time of bite**	**Number**	**%**
		**Number**		**Number**				
Hump-nosed pit viper (*Hypnalehypnale)*	8	5	63	3	37	Walking (outdoor)	3	37.5
						Playing (outdoor)	1	12.5
						Gardening	1	12.5
						Sleeping (Indoor)	2	25
						Playing (Indoor)	1	12.5
Russel’s viper (*Daboiarusselii)*	5	4	80	1	20	Walking(outdoor)	4	80
						Trying to wear the shoe (Indoor)	1	20
Saw-scaled viper *(Echiscarinatus*)	1	1	100	-	-	Walking	1	100
Cobra (*Najanaja)*	1	1	100	-	-	Walking	1	100
Common krait (*Bungaruscareuleus)*	3	-	-	3	100	Sleeping	2	67
						Playing	1	33

## Discussion

Out of the snake bite victims admitted to tertiary level hospitals in Bangladesh, 54% were bitten by nonvenomous snakes and 46% were bitten by venomous snakes [22]. According to the findings in our study, the mild and nonvenomous snake species accounted for 18% of the bites, and the venomous snakes accounted for 41% of the bites. Another 41% of the snakes that caused bites were not identified (Table 1). The prevalence of venomous snake bites in our study was similar to the findings of the study done in Bangladesh.

According to a study done in North India, 60% of bites were caused by elapids [8]. In our study, 32% of bites were caused by vipers and 9% of bites were caused by elapids (Table 1). The species of snakes and their venoms can vary from country to country and also vary within the same country.

In the present study bites occurred in the lower limbs, upper limbs, face and scalp regions in 73%, 20%, 5% and 2% of victims respectively (Table 3). Similar observations have been made in Bangladesh. The lower limbs and upper limbs have been bitten in 71% and 27% of victims respectively [7]. A previous study done in Jaffna peninsula Sri Lanka in 2011 mentions that 31% of patients were bitten on the fingers [20]. A study done in North Indian hospital have demonstrated that upper limbs were bitten in (47%) of victims [8]. In the present study the victims sustaining bites in the region of upper limbs is lower than the previous two studies done in Sri Lanka and North India.

The patients bitten on the face and scalp region is not described in many previous studies done in Sri Lanka and other countries in the world. However a study done in North India has observed that 7% of bites occur in the region of head and neck [8]. The finding of this study is similar to the finding of our study with regard to victims sustaining bites in the region of head and neck.

In North India, most of the bites (61%) occurred while patients were asleep (8). Indoor bites in our study accounted for 7 (16%) of the bites and out of those bites 4 (57%) bites occurred while sleeping on the floor (Table 6). Sleeping on the floor exposes the people to the bites of nocturnal snakes [6]. In our study nocturnal bites while sleeping were caused by common kraits (*Bungarus careuleus*) and Hump-nosed pit vipers (*Hypnale hypnale*).

The Asian cobra (*Naja naja*) bites typically occur outdoors in late afternoons [6]. In this study only one victim was bitten by a cobra (*Naja naja*) between 6 pm and 6 am while walking outdoors (Table 5). The most common signs were swelling of the affected limb and neurological features such as ptosis. Treatment with anti-venom and surgical debridement was necessary.

In Anuradhapura district of Sri Lanka, the highest (73%) number of snake bites is due to Russell’s viper (*Daboia russelii*) bites [6]. In the present study, Russell’s vipers (*Daboia russelii*) caused the second highest number of venomous bites 5 (12%) (Table 1). According to previous Russell’s viper (*Daboia russelii*) envenomation studies done in Sri Lanka shows that patient’s were affected with local swelling (92%), neurotoxicity (78%), coagulopathy (77%), nephrotoxicity (18%) and local necrosis (14%) [14]. Apart from local swelling and coagulopathy, other signs were less common in our study. In this study, the majority of Russell’s viper (*Daboia russelii*) bites (80%) occurred between 6 pm and 6 am while walking outdoors (Table 5) and antivenom had to be given to 80% of patients (Table 2).

Hump-nosed pit viper (*Hypnale hypnale*) accounted for highest number of snake bites in Sri Lanka and the patients experienced local swelling (80%), systemic symptoms (16%) and coagulopathy (3%) [6]. They reported the lower limbs were the most common bitten site and the majority of the bites occurred during day-time [23]. According to findings of our study, majority of bites occurred during the day-time and the lower limbs were the most common bitten site (Table 5). Swelling of the affected limb was the most common presenting sign. Our findings were similar to the previous study done by Maduwage et al. [23].

Local swelling and bleeding problems were the most common symptoms found and the lower limbs were the most common bitten site in our study and previous studies done on saw-scaled viper (*Echis carinatus*) bites in Sri Lanka, India and West Africa . Most saw-scaled viper (*Echis carinatus*) bites (69%) occurred in the night, outdoors [19]. However a study done in Jaffna peninsula Sri Lanka shows most saw-scaled viper (*Echis carinatus*) bites occurred during daylight [20]. In the present study, the victim was bitten between 6 pm and 6 am outdoors.

Kraits (*Bungarus* species) are known to bite their victims indoors during night time while asleep. Case fatalities such as respiratory paralysis can range from 77-100% without treatment [6]. In our study, 67% of the common krait (*Bungarus careuleus*) bites occurred indoors while sleeping (Table 6) and even after reaching the teaching hospital, deaths occurred in 67% of the cases. Bite time and symptoms reported in our study were similar to the findings of previous studies done on Kraits (*Bungarus* species) in South Asia. However, the deaths reported in our study were higher than the deaths reported in a previous Sri Lankan study done in North Central and North Western provinces of Sri Lanka [10].

Not many studies describe the first aid given to snake bite victims. The few studies that describe the first aid given to snake bite victims in Nepal, Bangladesh, Southern and Northern India show that accepted first aid guidelines were not followed [6]. In the present study, reassurance and immobilization has been followed in 80% and 75% cases respectively and unaccepted first aid measures such as application of herbal medicines and tourniquet have been done in 5% and 9% of cases respectively (Table 4). Tourniquets have been reportedly used in 90% and 98% of victims in Nepal and Bangladesh respectively [6]. In Bangladesh and India, it has been reported that incisions are made in and around the bite site in 42% and 20% of cases respectively [6]. Fortunately, incisions were not been made in any of the patients in our study. Many previous Sri Lankan studies have not published data with regards to the first aid measures used. According to the results of our study, the majority of subjects had been subjected to the accepted first aid measures compared to the findings from Nepal, Bangladesh and India.

According to a study done in Bangladesh, antivenom was given to 91% of venomous bites [22]. In our study, 18 (41%) were venomous bites and 7 (39%) required anti venom (Table 2). The severity of envenomation can vary from species to species and the life styles of the snakes. These can vary from country to country as well. In the year 2000, 0.5% of snake bite victims that were admitted to hospitals in Sri Lanka resulted in death [10]. In the present study, deaths occurred in 5% of bite victims. Patients who could not be saved were bitten by common kraits (*Bungarus careuleus*). Envenoming bites can give rise to respiratory failure within 30 minutes of the bite [6]. In our study the majority (82%) of bite victims had taken 1 hour or more to reach the teaching hospital (Table 4). A North Indian study has demonstrated that the median time to arrive at the hospital after a bite was 9 hours [8]. Differences in time between the two countries could be due to the differences in the availability of transport and the distance to hospital facilities.

## Conclusions

This study describes the features of five venomous snake species with regards to the site, time, place of bite and clinical features of bite victims. Studies done purely on paediatric snake bite victims are sparse. Even the studies done on adult snake bite victims, show that the majority of studies only describe features of one venomous snake with regard to the site, time, place of bite and clinical features. Of all the venomous bites in our study the Hump-nosed pit viper (*Hypnale hypnale*) accounted for the highest number, and the Russell’s viper (*Daboia russelii*) accounted for the second highest number. The majority of the bites occurred between 6 pm and 6 am, and the foot was the most common bitten site as most bites occurred while walking at night. A significant number of venomous bites occurred indoors while sleeping. The majority of the severely envenomated patients required antivenom. Findings of this study will be useful for doctors and other health workers in educating their patients and the general public on prevention and management of paediatric cases of venomous snake bite in Sri Lanka.

## Competing interests

There are no financial or non- financial competing interests to be declared with regard to the research work involved in this publication. RK and DMR are employed in the Ministry of Health and AL is employed in the Ministry of Higher Education. Author’s personal salaries provide for research. Their employers’ do not play a role in the design, data collection, analysis, interpretation, writing and decision to submit the manuscript.

## Authors’ contributions

RK was involved in conception and designing the study, data collection, patient management, data analysis and helping in drafting the manuscript. DMR was involved in patient management, conception and designing the study. AL was involved in conception and designing the study, data analysis, interpretation of data, drafting the manuscript and revising it critically. All the authors read and approved the final manuscript.
